# Prognostic significance of helicobacter pylori-infection in gastric diffuse large B-cell lymphoma

**DOI:** 10.1186/s12885-019-6067-5

**Published:** 2019-08-28

**Authors:** Yuan Cheng, Yinan Xiao, Ruofan Zhou, Yi Liao, Jing Zhou, Xuelei Ma

**Affiliations:** State Key Laboratory of Biotherapy and Cancer Center, West China Hospital, Sichuan University and Collaborative Innovation Center, Chengdu, People’s Republic of China

**Keywords:** Helicobacter pylori, Stomach, Diffuse large B-cell lymphoma, Prognosis, Survival analysis

## Abstract

**Background:**

Helicobacter pylori (*H. pylori*) is thought to have an oncogenic effect on the development of gastric malignancies. However, the effect of *H. pylori* status on the prognosis of gastric diffuse large B-cell lymphoma (DLBCL) remains unconfirmed. This study aimed to identify the prognostic importance of *H. pylori* infection in de novo gastric DLBCL.

**Methods:**

One hundred and twenty-nine patients diagnosed with primary de novo gastric DLBCL at the West China Hospital of Sichuan University from 1st January 2009 to 31st May 2016 were included. The clinical features of the patients were documented. *H. pylori* status was assessed via urease breath tests and histologic examinations. The prognostic value of *H. pylori* was verified via univariate and multivariate analyses.

**Results:**

Over a median follow-up of 52.2 months (range 4–116), the 5-year overall survival (OS) for all patients was 78.7%. Patients with *H. pylori* infections had significantly better 5-year PFS and OS than did the *H. pylori*-negative subgroup (5-year PFS, 89.3% vs. 74.1%, *P* = 0.040; 5-year OS, 89.7% vs. 71.8%, *P* = 0.033). Negative *H. pylori* status and poor ECOG performance were independent negative prognostic indicators for both PFS and OS (PFS, *P* = 0.045 and *P* = 0.001, respectively; OS, *P* = 0.021 and *P* < 0.001, respectively).

**Conclusions:**

*H. pylori* status in de novo gastric DLBCL can be a promising predictor of disease outcome, and patients with negative *H. pylori* status require careful follow-up since they tend to have a worse outlook.

## Background

The gastrointestinal tract is a common site of extranodal non-Hodgkin lymphoma (NHL), with the stomach being the affected site in 60% of all NHL patients with digestive tract involvement [1, 2]. Among gastric lymphomas, mucosa-associated lymphoid tissue (MALT) lymphoma and diffuse large B-cell lymphoma (DLBCL) are the two most common types [3]. DLBCL in the stomach is a heterogeneous disease, and it is usually divided into two categories: DLBCL with features of MALT lymphoma (DLBCL (MALT)) and DLBCL without evidence of MALT (de novo DLBCL or pure DLBCL) [4]. Up to 20% of patients with DLBCL have been identified as a concurrent component of MALT [5].

Helicobacter pylori (*H. pylori*), a spiral-shaped, micro-aerophilic bacterium that inhabits the human stomach, is estimated to colonize more than half of the world’s human population [6]. The bacterium has been categorized as a class I carcinogen, giving rise to a new method for classifying gastric carcinoma [7]. Previous studies showed that *H. pylori* infections can induce a gastric lymphoid tissue response and that it might be an oncogenic factor during the development of malignant gastric lymphomas, including MALT lymphoma and DLBCL [8–10]. However, accumulating evidence has proven that *H. pylori*-positive status can support long-term survival and lead to better prognoses in gastric carcinoma patients [11–13]. It is thought that DLBCL (MALT) is independent of *H. pylori* status, as it fails to respond to antibiotic therapy according to the WHO (World Health Organization) classification and contains a component of high-grade transformed MALT lymphoma that differs from low-grade and *H. pylori*-dependent MALT lymphomas (MALT lymphoma) [5, 14–16]. However, numerous studies have found that a substantial percentage of gastric DLBCL (MALT) is associated with *H. pylori* infection and that it responds effectively to *H. pylori* eradication, especially early-stage DLBCL (MALT) [17, 18]. Of note, de novo DLBCL, the pathogenesis of which was once thought to be different from that of MALT lymphoma, has also been proven to correlate with *H. pylori* status based on limited data [19, 20]. Until now, this clinically relevant finding has not been validated in large studies at other medical centers.

In this retrospective study, we evaluated the prognostic value of *H. pylori* status for patients with de novo DLBCL, especially among the early-stage population, at a larger medical center.

## Methods

### Patients: diagnosis and treatment

**Fig. 1:**
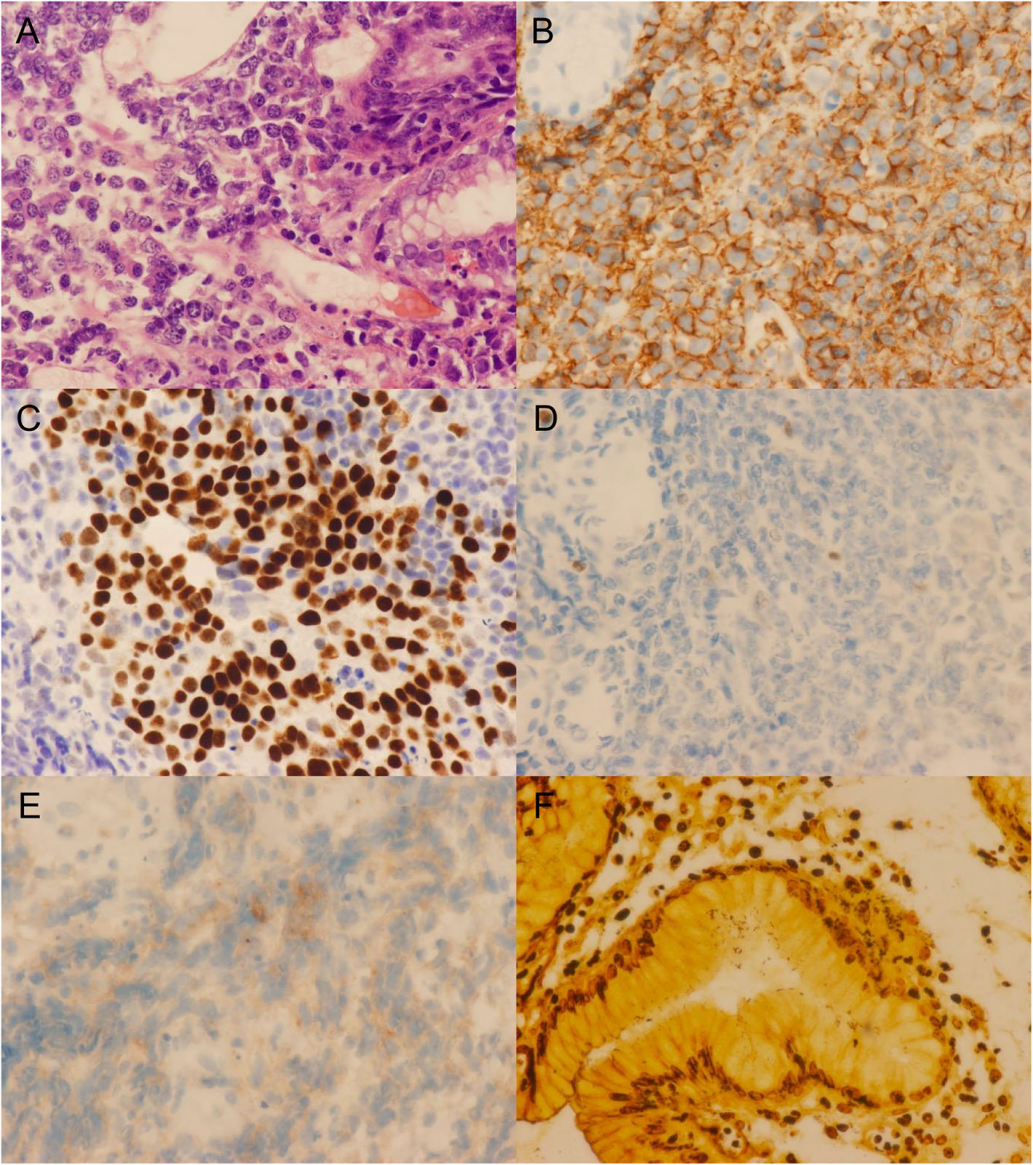
An example of immunohistochemical features of germinal center B-cell de novo gastric DLBCL. **a** diffuse large cells infiltrating the mucosa (hematoxylin-eosin (H&E) staining, × 400), (**b**) positive CD20 expression, (**c**) positive BCL6 expression, (**d**) negative expression of CD10, (**e**) negative expression of MUM1, and (**f**) Warthin-Starry staining of *H. pylori*

A retrospective review of all patients with the diagnosis of primary de novo DLBCL of the stomach at West China Hospital of Sichuan University from 1st January 2009 to 31st May 2016 was performed. Pathological specimens were obtained from both endoscopic biopsies and surgical resections, and diagnosis was based on the World Health Organization (WHO) classification system for hematologic malignancies [16]. Tumors without histological features of MALT lymphoma, including dense infiltration of centrocyte-like cells in the lamina propria and typical lymphoepithelial lesions [21, 22], were classified as de novo DLBCL. Only patients with primary involvement of stomach or with predominant gastric lesions were included. Patients with secondary gastric lymphoma or evidence of MALT origin were excluded. Paraffin-embedded, formalin-fixed tumor specimens where immunohistochemically stained for CD20, CD3, CD5, CD10, BCL6 and MUM1 (Fig. 1).

Patients were treated with the following therapeutic modalities singly or in combination: surgery, chemotherapy or radiotherapy. Chemotherapy referred to CHOP (cyclophosphamide, doxorubicin, vincristine and prednisone) or CHOP-like regimens, with or without rituximab (R).

### *H. pylori* infection

The status of *H. pylori* infection was confirmed at the baseline for each case via the results of at least one of two tests: histologic examination or a ^14^C-urea breath test (^14^C -UBT). The histologic examination consisted of Warthin-Sharry (W-S) staining of tissue specimens. The results of the ^14^C-UBT were reported as ^14^C disintegrations per minute (dpm), and dpm ≥ 100 and dpm < 100 were interpreted as positive and negative, respectively. Of note, all *H. pylori*-positive patients received antibiotic therapy against *H. pylori*, including bismuth compounds, proton pump inhibitor (PPI) and two of the following antibiotics: clarithromycin, amoxicillin, metronidazole or tetracycline.

### Clinical evaluation

Staging workups included the collection of a detailed medical history, a physical examination that included examination of the peripheral lymph nodes and Waldeyer’s ring, complete hematological biochemical examinations, including serum lactate dehydrogenase (LDH), computed tomography (CT), gastric endoscopy, endoscopic ultrasonography, positron emission tomography (PET)/CT, and bone marrow aspiration and biopsy. For patients who received surgical interventions, the intraoperative exploration and pathological results were further considered. The staging and classification of the lesions were based on the Lugano staging system [23]. Furthermore, the performance status of each patient was assessed according to the Eastern Cooperative Oncology Group (ECOG) scale and the International Prognostic Index (IPI).

### Statistical analyses

Pearson’s chi-squared test and Fisher’s exact test were used to evaluate the relationships between *H. pylori* status and other prognostic indicators and clinicopathological characteristics. Analyses were performed using follow-up data available on 31st May 2018. The primary endpoints of the current study were progression-free survival (PFS) and overall survival (OS). PFS was defined as the date of initial diagnosis until disease progression, relapse or death due to any cause. OS was measured from the date of diagnosis to the date of death from any cause or the date of a survivor’s final follow-up. Survival curves were estimated via the Kaplan-Meier method, and the differences between survival curves were compared via the log-rank test. All of the significant variables in the univariate analyses were included in multivariate analyses to evaluate the independent risk factors for PFS and OS via the Cox proportional hazards regression model. SPSS version 21.0 was used for the data analyses. *P*-values less than 0.05 were considered statistically significant. Our team has reviewed the data and reached an agreement on this final version.

## Results

### Baseline characteristics

The demographic baseline data and clinicopathologic parameters of the 129 included patients with de novo gastric DLBCL are listed in Table 1. The median age was 55 years old with a range of 21–84. 66 (51.2%) of the patients were women. Among the 129 patients, 64 (49.6%) were negative for *H. pylori* and 65 (50.4%) were positive for *H. pylori*. 71 (86.6%) patients had lesions in the antrum or corpus of the stomach, and B symptoms appeared in 45 (34.9%) patients. Most patients (> 50%) were early-stage and had better performance based on Lugano stage, IPI score and ECOG scale. Of note, the distribution of Lugano stage, IPI risk group, resection surgical treatment and LDH level were significantly associated with the *H. pylori*-positive and -negative subgroups (*P* < 0.05). Patients with *H. pylori* infections tended to remain in an early-stage of de novo DLBCL, whereas patients in the *H. pylori*-negative subgroup had relatively poor performance. The remaining clinical variables were similar in both *H. pylori* subgroups (*P* > 0.05).

**Table 1:** Association between clinicopathologic features and *H. pylori* status of 129 patients with de novo gastric diffuse large B-cell lymphoma

Patients	Number of patients *n* = 129(%)	*H. pylori*-negative *n* = 64 (%)	H. pylori-positive *n* = 65 (%)	*p*
Gender				
Female	66(51.2)	39(53.4)	27(48.2)	0.557
Male	63(48.8)	34(46.6)	29(51.8)	
Age, years				
< 60	73(56.6)	43(58.9)	30(53.6)	0.545
≥ 60	56(43.4)	30(41.1)	26(46.4)	
Presence of B symptoms				
No	84(65.1)	43(58.9)	41(73.2)	0.091
Yes	45(34.9)	30(41.1)	15(26.8)	
Tumor sites				
Proximal	11(13.4)	7(17.1)	4(9.8)	0.331
Distal	71(86.6)	34(82.9)	37(90.2)	
Lugano stage				
I/II	88(66.7)	44(60.3)	44(78.6)	**0.027**
IIE/IV	41(33.3)	29(39.7)	12(21.4)	
IPI risk group				
Low (intermediate) risk	109(84.5)	57(78.1)	52(92.9)	**0.022**
High (intermediate) risk	20(15.5)	16(21.9)	4(7.1)	
ECOG				
0–1	117(90.7)	65(89.0)	52(92.9)	0.460
≥ 2	12(9.3)	8(11.0)	4(7.1)	
Surgical treatment				
No	87(67.4)	43(58.9)	44(78.6)	**0.018**
Yes	42(32.6)	30(41.1)	12(21.4)	
Histological analyses				
non-GCB	56(70.0)	28(66.7)	28(73.7)	0.494
GCB	24(30.0)	14(33.3)	10(26.3)	
LDH				
< 220 U/L	92(71.3)	44(60.3)	48(85.7)	**0.002**
≥ 220 U/L	37(28.7)	29(39.7)	8(14.3)	

The *P*-values with statistical significance are shown in bold

Over a median follow-up of 52.2 months (range 4–116), the 5-year OS for all patients was 78.7%. The overall median PFS was 91.8 months (95% confidence interval [CI] 83.8–99.7), and the median overall survival (OS) was 96.0 months (95% CI 88.9–103.0). Patients with *H. pylori*-positive de novo gastric DLBCL had significantly better 5-year PFS and OS than patients in the *H. pylori*-negative subgroup (5-year PFS, 89.3% vs. 74.1%, *P* = 0.040; 5-year OS, 89.7% vs. 71.8%, *P* = 0.033) (Tables 2 and 3).

**Table 2:** Univariate and multivariate analysis of prognostic factors for progression-free survival in 129 patients with de novo gastric diffuse large B-cell lymphoma

Patients	Number of patients *n* = 129	Number of event *n* = 28	Median PFS	95% CI	Actuarial 5-year progression-free survival (%)	Univariate analysis		Multivariate analysis	
						HR (95% CI)	*p*	HR (95% CI)	*p*
Gender									
Female	66	15	79.2	70.1–88.3	78.0	1	0.691	–	
Male	63	13	94.0	83.4–104.6	81.8	0.860(0.409–1.809)		–	
Age, years									
< 60	73	12	96.4	86.9–105.9	87.7	1	**0.098**	1	0.871
≥ 60	56	16	85.6	73.1–98.1	70.1	1.886(0.889–4.003)		1.078(0.435–2.671)	
Presence of B symptoms									
No	84	13	99.5	91.3–107.6	86.5	1	**0.012**	1	**0.004**
Yes	45	15	66.3	54.8–77.7	68.1	2.605(1.235–5.493)		3.337(1.466–7.595)	
Tumor sites									
Proximal	11	4	55.0	34.8–75.2	49.1	1	0.213	–	
Distal	71	16	91.1	80.5–101.8	78.9	0.495(0.164–1.495)		–	
Lugano stage									
I/II	88	14	98.2	89.6–106.8	85.1	1	**0.013**	1	0.666
IIE/IV	41	14	66.4	53.4–79.3	69.1	2.573(1.222–5.417)		1.129(0.651–1.960)	
IPI risk group									
Low (intermediate) risk	109	17	102.1	95.3–108.9	85.3	1	**< 0.001**	1	0.117
High (intermediate) risk	20	11	58.5	39.4–77.6	50.0	4.364(2.033–9.366)		2.924(0.764–11.186)	
ECOG									
0–1	117	19	97.8	90.3–105.4	85.3	1	**< 0.001**	1	**0.001**
≥ 2	12	9	30.7	12.0–49.3	27.8	8.190(3.644–18.407)		5.002(1.970–12.698)	
Surgical treatment									
No	87	22	68.1	61.0–75.2	76.0	1	**0.044**	1	0.068
Yes	42	6	102.0	91.7–112.4	87.6	0.384(0.152–0.974)		0.381(0.135–1.075)	
Histological analyses									
non-GCB	56	11	77.0	67.9–86.1	85.6	1	0.702	–	
GCB	24	6	76.6	62.8–90.5	70.5	1.214(0.448–3.290)		–	
Lactate dehydrogenase									
< 220 U/L	92	16	96.6	87.9–105.3	82.9	1	**0.059**	1	0.646
≥ 220 U/L	37	12	69.8	56.7–82.9	72.8	2.059(0.973–4.357)		1.108(0.716–1.713)	
H. pylori status									
Negative	73	22	85.0	74.2–95.7	74.1	1	**0.040**	1	**0.045**
Positive	56	6	100.6	92.0–109.2	89.3	0.388(0.157–0.959)		0.379(0.147–0.978)	

The *P*-values with statistical significance are shown in bold

**Table 3:** Univariate and multivariate analysis of prognostic factors for overall survival in 129 patients with de novo gastric diffuse large B-cell lymphoma

Patients	Number of patients *n* = 129	Number of death *n* = 25	Median OS	95% CI	Actuarial 5-year overall survival (%)	Univariate analysis		Multivariate analysis	
						HR (95% CI)	*p*	HR (95% CI)	*p*
Gender									
Female	63	14	81.7	73.3–90.2	76.9	1	0.522	–	
Male	66	11	98.5	89.1–107.8	80.3	0.773(0.351–1.702)		–	
Age, years									
<60	73	9	102.2	94.4–110.0	85.6	1	**0.033**	1	0.525
≥ 60	56	16	86.8	74.9–98.8	69.9	2.434(1.075–5.511)		1.384(0.507–3.779)	
Presence of B symptoms									
No	84	13	99.9	91.9–107.9	84	1	**0.139**	1	0.094
Yes	45	12	76.3	65.8–86.8	68.9	1.810(0.825–3.969)		2.129(0.879–5.154)	
Tumor sites									
Proximal	11	4	60.2	44.4–75.9	60.6	1	0.212	–	
Distal	71	14	95.6	86.1–105.1	76.4	0.492		–	
Lugano stage									
I/II	88	12	102.1	94.9–109.4	85.3	1	**0.012**	1	0.667
IIE/IV	41	13	70.3	57.7–82.7	66.6	2.756(1.251–6.070)		1.292(0.402–4.153)	
IPI risk group									
Low (intermediate) risk	109	15	102.0	95.4–108.5	83.7	1	**< 0.001**	1	0.256
High (intermediate) risk	20	10	55.1	37.2–72.9	51.4	5.088(2.263–11.442)		2.242(0.556–9.035)	
ECOG									
0–1	117	16	102.1	95.8–108.4	83.7	1	**< 0.001**	1	**< 0.001**
≥ 2	12	9	33.3	16.6–50.1	31.3	9.741(4.249–22.336)		6.216(2.391–16.159)	
Surgical treatment									
No	87	21	69.9	63.2–76.6	73.7	1	**0.021**	1	**0.030**
Yes	42	4	107.9	100.4–115.5	89.4	0.281(0.095–0.827)		0.273(0.084–0.881)	
Histological analyses									
non-GCB	56	10	80.4	72.3–88.6	78.5	1	0.873	–	
GCB	24	5	80.9	68.3–93.5	76.9	0.957(0.559–1.637)		–	
Lactate dehydrogenase									
< 220 U/L	92	15	99.1	91.3–106.9	81.7	1	**0.134**	1	0.885
≥ 220 U/L	37	10	75.4	63.4–87.3	71.5	1.845(0.828–4.108)		1.065(0.451–2.517)	
H. pylori status									
Negative	73	20	89.3	79.4–99.3	71.8	1	**0.033**	1	**0.021**
Positive	56	5	102.7	94.9–110.5	89.7	0.344(0.129–0.917)		0.292(0.103–0.828)	

The *P*-values with statistical significance are shown in bold

### Univariate and multivariate analyses

We next investigated the associations between important clinicopathologic parameters and patient survival via a Cox proportional hazard regression analysis (Tables 2 and 3).

Upon follow-up, 28 patients showed disease progression (22 were *H. pylori*-negative and 6 were *H. pylori*-positive). The mean PFS was 100.6 months (95% CI 92.0–109.2) in patients positive for *H. pylori*, compared with 85.0 months (95% CI 74.2–95.7) for patients in the *H. pylori*-negative subgroup (*P* = 0.040). The presence of B symptoms (*P* = 0.012), advanced Lugano stage (*P* = 0.013), poor ECOG performance status (*P* < 0.001), classification into higher IPI risk groups (*P* < 0.001), lack of resection surgery (*P* = 0.044) and negative *H. pylori* status (*P* = 0.040) were significantly associated with poor PFS of de novo gastric DLBCL in the univariate analyses (Table 2). The multivariate analyses revealed that the presence of B symptoms (hazard ratio = 2.605; *P* = 0.004) and poor ECOG performance status (hazard ratio = 5.002; *P* = 0.001) could independently predict poor PFS outcomes of de novo gastric DLBCL. However, positive *H. pylori* status was a positive predictor of PFS (hazard ratio = 0.379; *P* = 0.045) (Table 2).

Among the 25 patients who died during the follow-up, 5 were *H. pylori*-positive and the remained were *H. pylori*-negative. The mean OS was 102.7 months (95% CI 94.9–110.5) in *H. pylori*-positive patients, compared with 89.3 months (95% CI 79.4–99.3) in the patients in the *H. pylori*-negative subgroup (*P* = 0.033). Univariate analyses showed significant associations between OS and age (P = 0.033), Lugano staging (*P* = 0.012), ECOG performance (*P* < 0.001), IPI risk group (P < 0.001), resection surgery (*P* = 0.021), and *H. pylori* infection (*P* = 0.020) (Table 3). Multivariate analyses revealed that ECOG performance (hazard ratio = 6.216; *P* < 0.001) was an independent prognostic index for poor OS in de novo gastric DLBCL. Resection surgery (hazard ratio = 0.273; *P* = 0.003) and *H. pylori* infection (hazard ratio = 0.292; P = 0.021) were positive prognostic factors for OS (Table 3).

### Kaplan-Meier survival analysis

**Fig. 2:**
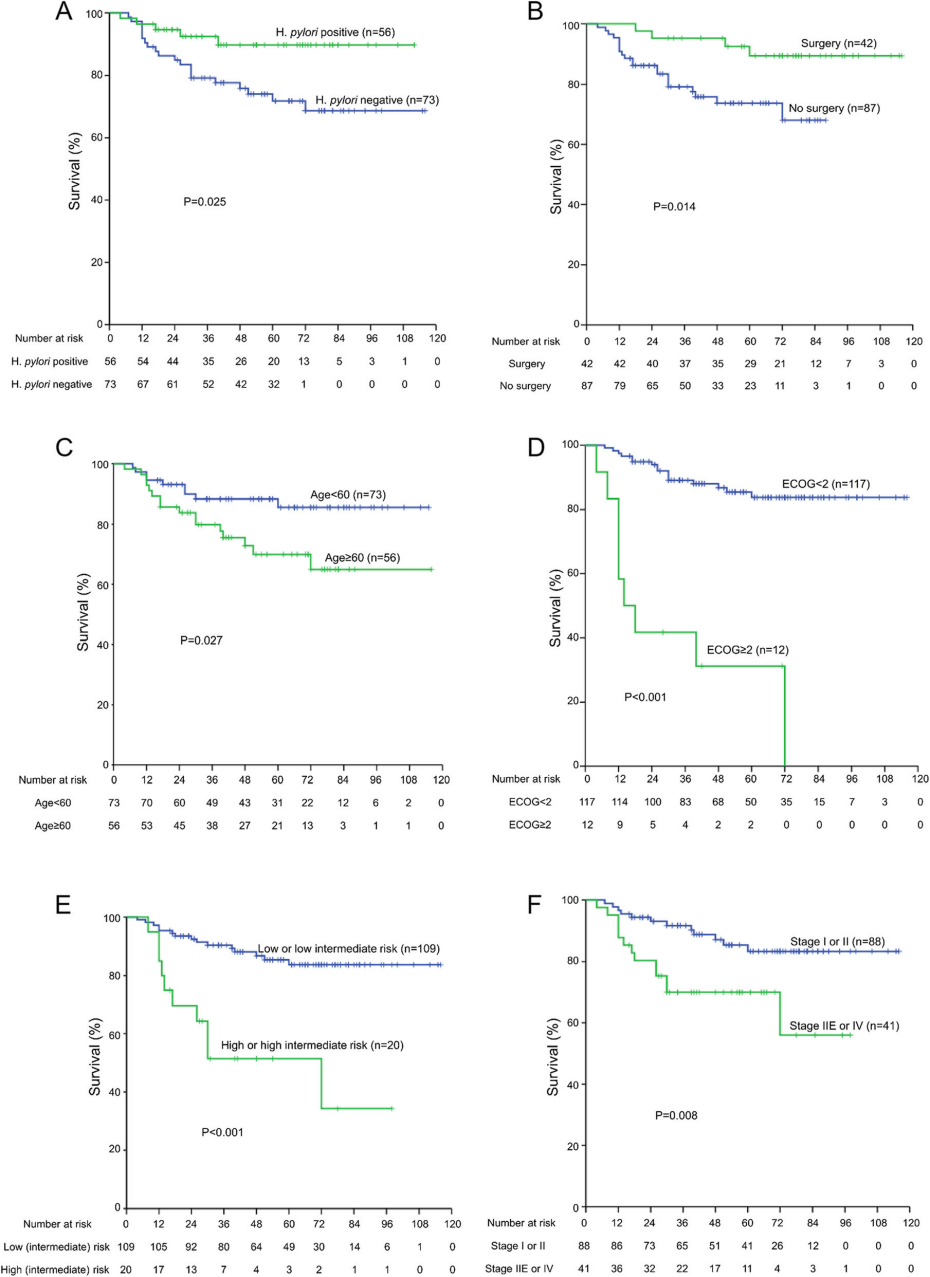
Effects of *H. pylori* status, surgery, age, ECOG performance, IPI risk group, and Lugano stage on overall survival of patients with de novo gastric diffuse large B-cell lymphoma according to Kaplan-Meier analysis. **a** Positive *H. pylori* status (*p* = 0.025), (**b**) Surgery (*p* = 0.014), (**c**) Age < 60 (*p* = 0.027), (**d**) ECOG< 2 (*p* < 0.001), (**e**) low and low intermediate risk (p < 0.001) and (**f**) Lugano stage I and II (*p* = 0.008) are positive prognostic factors for de novo gastric DLBCL

Kaplan-Meier survival curves showed that *H. pylori* infection status (*P* = 0.025), resection surgery (*P* = 0.014), age (*P* = 0.027), ECOG performance status (P < 0.001), IPI risk group (P < 0.001) and Lugano stage (*P* = 0.008) were significantly associated with OS of de novo DLBCL (Fig. 2).

### Subgroup analysis

**Fig. 3:**
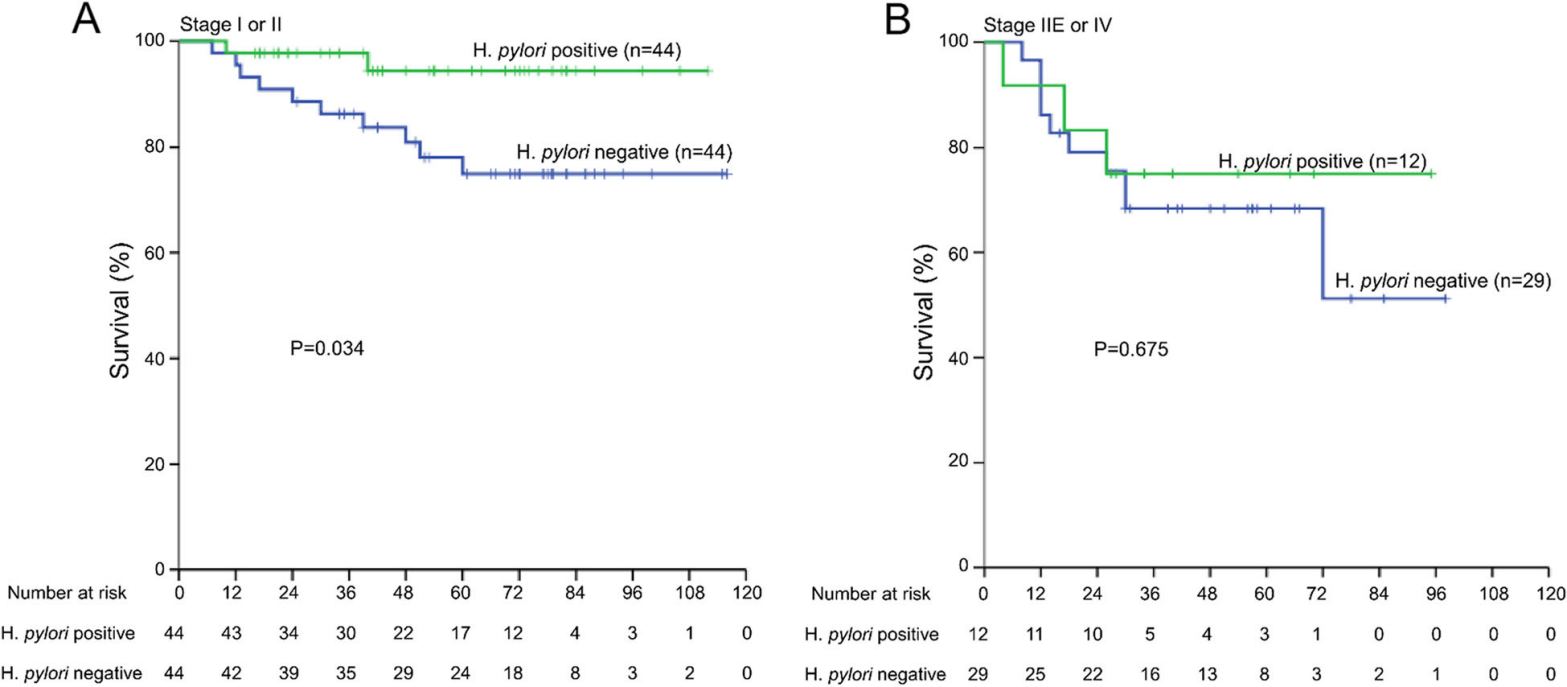
Effects of *H. pylori* status according to Lugano stage on overall survival of patients with de novo gastric diffuse large B-cell lymphoma. Positive *H. pylori* status is associated with better prognosis in patients of (**a**) Lugano stage I and II rather than those of (**b**) stage IIE and IV (*p* = 0.034 and *p* = 0.675, respectively)

Of note, in stage-specific analyses, *H. pylori* infection presented a significant association with OS in stage I or II (*P* = 0.034) but not in stage IIE or IV (*P* = 0.675) (Fig. 3).

## Discussion

Gastric MALT lymphoma has been claimed to be strongly associated with *H. pylori* infection, and eradication of *H. pylori* (HPE) even without oncological treatment has been used as the first-line treatment for this disease, especially for early stage MALT lymphoma [24, 25]. Gastric DLBCL (MALT) results from high-grade transformation of MALT lymphoma and is independent of *H. pylori* infection; however, previous studies have also proven that *H. pylori* eradication can lead to complete remission in a certain fraction of patients with *H. pylori*-positive DLBCL (MALT) [26]. Researchers have also found that some de novo DLBCL patients also presented complete remission after *H. pylori* eradication [27, 28]. Furthermore, an association between *H. pylori* infection and de novo DLBCL was validated in a large cohort [29]. Our results suggested that *H. pylori*-positive status was strongly indicative of better gastric de novo DLBCL prognosis.

*H. pylori*-induced lymphomagenesis is a multistep process involving *H. pylori* virulence factors (e.g., CagA, VacA and OipA), host factors and environmental conditions. Cytotoxin-associated gene A (CagA) protein, which is exposed on the surface of *H. pylori* cells via a type IV secretion system, is the most extensively studied *H. pylori* virulence factor. CagA can directly pass through the host membrane via an interaction with phosphatidylserine, after which it perturbs cell signaling in a way that can lead to oncogenesis [30]. Consistent with previous studies, our results indicated that despite the carcinogenic effect of *H. pylori*, *H. pylori* infection was associated with a less aggressive subtype of de novo DLBCL, and these patients showed better prognoses [19, 20]. Further subgroup analyses suggested that *H. pylori* infection was significantly associated with better survival outcomes in patients with early-stage gastric de novo DLBCL. As the identification of *H. pylori* status is possible during early cancer stages, it is useful for stratifying patients into risk groups and for predicting adverse disease outcomes; thus, its identification of as an independent predictor could establish it as a promising tool in clinical practice for helping to make treatment decisions.

One possible explanation for the critical role of *H. pylori* infection in improving the outlook of gastric de novo DLBCL is the immune cross-reactivity between *H. pylori* and the malignant B-cells of gastric DLBCL. Furthermore, previous studies have hypothesized that antigenic mimicry between *H. pylori* and the gastric mucosa might result in immune cross-reactions that affect tumor cells and suppress tumor progression as well as metastasis in gastric carcinoma [31, 32]. Furthermore, the immune response induced by *H. pylori* might also be cross-reactive against the malignant B-cells of gastric DLBCL since *H. pylori* can transfer CagA into both epithelial cells and B-lymphocytes and promote CagA expression in malignant B-cells [33]. Immune cross-reactions with malignant cells are characterized by the presence of mimic or absorbed *H. pylori* antigens, which results in improved survival outcomes in *H. pylori*-positive gastric DLBCL.

These newly uncovered underlying mechanisms demonstrate that *H. pylori*-positive de novo gastric DLBCL has less aggressive behavior. By examining genome-wide expression profiles of both mRNAs and miRNAs in *H. pylori*-positive and -negative de novo gastric DLBCL tissue specimens, a previous study confirmed that *H. pylori* infection is associated with a higher level of miR-200, which can inhibit Zinc-finger E-box-binding homeobox 1 (ZEB1) [20]. ZEB1 is expressed at a significantly higher level in DLBCL than that in reactive lymphoid tissue and is linked to an adverse prognosis in DLBCL [34]. ZEB1 promotes DLBCL progression via downregulation of BCL6, which is a known positive predictor for DLBCL [20]. However, the specific role of BCL6 in DLBCL progression requires further investigation. In light of its role in carcinogenesis, *H. pylori* might also affect other biological behaviors of gastric DLBCL, which may contribute to the survival benefits observed in the *H. pylori*-positive subgroup. Inherent differences in the tumors that develop secondary to *H. pylori*-infection and the carcinogenic functions of *H. pylori* infection require additional study.

This study is limited by its retrospective nature and single-institution bias. Furthermore, not all patients included in this study were treated with rigorous standard therapy. Some of the patients received chemotherapy without rituximab due to economic pressure, while other patients underwent surgery because they were either diagnosed with gastric DLBCL after surgery, or they were diagnosed years ago when surgery was still commonly performed. Despite the aforementioned limitations, to our knowledge, this study is the largest report examining the prognostic role of *H. pylori* in de novo gastric DLBCL.

## Conclusions

In summary, patients with primary gastric de novo DLBCL without *H. pylori* infection are more likely to have poor prognoses than patients with the infection; therefore, the patients without *H. pylori* may benefit from more aggressive treatment and more systematic follow-up.

## Data Availability

All data generated or analysed during this study are included in this published article.

## Abbreviations

CagA: Cytotoxin-associated gene A
CEA: Carcinoembryonic antigen
DLBCL: Diffuse large B-cell lymphoma
ECOG: Eastern Cooperative Oncology Group
EFS: Progression-free survival
GCB: Germinal center B-cell
*H. pylori*: Helicobacter pylori
IPI: International Prognostic Index
LDH: Lactate dehydrogenase
MALT: Mucosa-associated lymphoid tissue
NHL: Non-Hodgkin’s Lymphomas
OS: Overall survival
